# Constraining Continuous Topology Optimizations to Discrete Solutions for Photonic Applications

**DOI:** 10.1021/acsphotonics.2c00862

**Published:** 2023-01-09

**Authors:** Conner Ballew, Gregory Roberts, Tianzhe Zheng, Andrei Faraon

**Affiliations:** Kavli Nanoscience Institute and Thomas J. Watson Sr. Laboratory of Applied Physics, California Institute of Technology, Pasadena, California91125, United States

**Keywords:** metaoptics, topology optimization, photonics

## Abstract

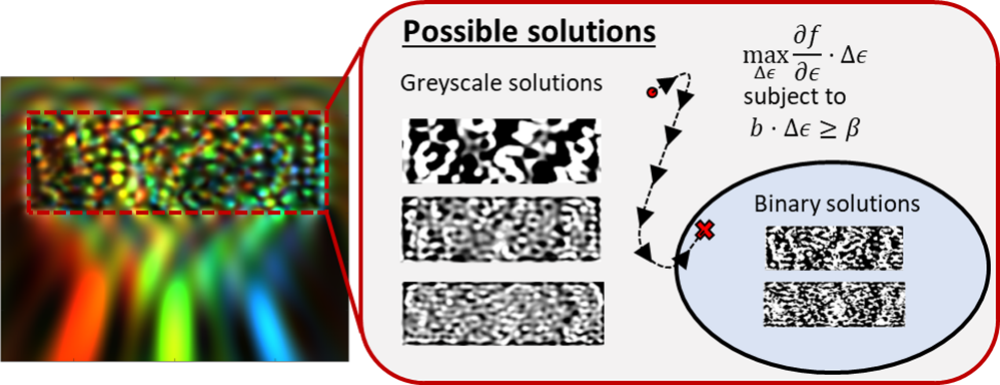

Photonic topology optimization is a technique used to find the permittivity distribution of a device that optimizes an electromagnetic figure-of-merit. Two common versions are used: continuous density-based optimizations that optimize a gray scale permittivity defined over a grid, and discrete level-set optimizations that optimize the shape of the material boundary of a device. In this work we present a method for constraining a continuous optimization such that it is guaranteed to converge to a discrete solution. This is done by inserting a constrained suboptimization with low computational overhead cost at each iteration of an overall gradient-based optimization. The technique adds only one hyperparameter with straightforward behavior to control the aggressiveness of binarization. Computational examples are provided to analyze the hyperparameter behavior, show this technique can be used in conjunction with projection filters, show the benefits of using this technique to provide a nearly discrete starting point for subsequent level-set optimization, and show that an additional hyperparameter can be introduced to control the overall material/void fraction. This method excels for problems where the electromagnetic figure-of-merit is majorly affected by the binarization requirement and situations where identifying suitable hyperparameter values becomes challenging with existing methods.

## 1 Introduction

Photonic inverse design is the process of choosing a figure-of-merit (FoM) and finding an optimal photonic device that maximizes or minimizes the FoM. A subset of photonic inverse design is topology optimization, which entails altering the shape of the photonic structure (i.e., its topology) to achieve the desired performance.^1^ In this case the device is characterized by its electric permittivity ϵ(*x*, *y*, *z*) in a three-dimensional (3D) design region.

A useful technique for inverse-design is gradient descent. This is the process of finding a local minimum of a function *f*(*x*) by following the gradient ∇*f*. The functions being minimized are generally nonlinear, thus a computed gradient is only valid in a locally linear region of *f*. Gradient descent is therefore an iterative optimization, comprised of computing ∇*f*, stepping *f* in the direction of −α∇*f* where α is a sufficiently small step size, and concluding the optimization when ∇*f* is sufficiently close to 0. A major enabling technique for gradient-based optimization is the adjoint method for efficiently computing a gradient. The technique has been applied to many fields (see ref (2) for a recent overview of the topic), including photonic design.^3−6^

The gradient-descent procedure becomes increasingly complex as the dimensions of the parameter space increase and constraints are imposed. For optimizing the topology of photonic devices, the parameter space includes the permittivity of every voxel in the design region, which can be arbitrarily large. For many photonic devices, the desired solution is constrained due to the limitations of available fabrication techniques that make only certain solutions physically realizable. Some common requirements of current fabrication techniques include the following:

- *Minimum feature size*: the size of the smallest material or void feature in the design, often quantified as a minimum gap size or radius of curvature.^7,8^ This is typically dictated by lithography, material etching, and material deposition capabilities for optical devices.
- *Connectivity*: one or more materials are topologically connected.^9^
- *Binary*: the device is made of only two materials.^8,10^

There are many topology optimization algorithms and many reviews summarizing them.^11^ They can be characterized as density-based optimization or shape optimization: density-based optimizations feature a density variable ρ that is evaluated across a grid of points, thus describing the device in an element-wise way. Density-based optimization can be further classified as discrete, where ρ = 0 or ρ = 1, or continuous, where 0 ≤ ρ ≤ 1. Shape-based optimizations instead describe the boundary of the device Ω. One common technique for shape-based optimizations involves describing the boundary Ω as the level-set contour of a higher-dimensional function, called the level-set method. A technique employed in photonic optimization begins with a density-based optimization, then switches to a level-set method to conclude the optimization.^4^ In this framework it can be beneficial for the density-based optimization to converge to a sufficiently high-performing and nearly binary starting point for the subsequent level-set method. This represents a challenge for devices that feature materials with large refractive index contrast, which will be shown in section 3 of this work.

The goal of binarizing a continuous density-based optimization is not new, and is not limited to photonic inverse-design. Commonly used methods for this task include projection filters, which encourage the device to become more binary but fall short of explicitly requiring the device become binary.^12^ Filters based on gradually strengthened sigmoidal or Heaviside filters have been used to aid convergence to a binary solution.^9,13,14^ Other methods to facilitate binarization include incorporating penalty terms for minimizing gray regions^10^ and incorporating loss into gray regions to discourage this material.^15^ However, these do not guarantee that the device becomes binary since the binarization constraint is not strongly enforced, which allows solutions to relax toward gray solutions.^16^ The final thresholding operation (in which all voxels are rounded to 0 or 1) can thus incur a severe performance penalty that requires substantial postprocessing to overcome, since this procedure ignores FoM gradient information. Furthermore, these techniques tend to involve multiple hyperparameters that have a substantial effect on the outcome of the optimization, and the optimal values of the hyperparameters often vary from problem to problem.

We present a method here that reformulates the gradient-based continuous density optimization in a manner that strictly enforces that the design become fully binary, thus preventing the optimization from settling at a nonbinary solution. The method uses the gradient information at each iteration to step the permittivity in a direction that maximizes the performance of the device while constraining the permittivity step to binarize the device by some amount that is greater than zero. We refer to this procedure as the “suboptimization”, since it occurs at every iteration of the overall photonic optimization. Some of the key advantages of this technique are the following:

- It requires only one hyperparameter that intuitively controls the aggressiveness of binarization (section 3.1).
- It can be used as a stand-alone method or integrated with projection filters (section 3.2).
- Another simple hyperparameter can be introduced to control the material/void fraction (section 3.3).
- The addition of the hard constraint adds very little computational overhead to the overall optimization (Supporting Information, section 2).

The remainder of the paper is composed as follows: first, we formulate the suboptimization and derive the solution of the Lagrange dual problem; then, we provide several computational examples of an optimized spectral demultiplexer. The examples illustrate the practical usage of the method, study the algorithm performance for different hyperparameter values, demonstrate the ability to include the proposed method with projection filters, integrate the technique with a subsequent level-set optimization, and describe a technique to control the overall fraction of material in the final device by introducing an additional hyperparameter to the technique.

## 2 Methods

### 2.1 Setup

Consider a discretized 3D domain of interest . The domain contains a region of dielectric permittivity , where  is called the “design region” or “device”. The FoM  depends on the electric and magnetic fields output from the device, and the permitivities vector of the design region  with components . The general goal of the inverse-design optimization is to find the optimal permittivity of the design region that maximizes , and here we wish to constrain the solutions to only permittivity distributions consisting of two materials. ϵ_min_ and ϵ_max_ are the lower and upper bounds of the permittivity, respectively, representing the two materials the final device is comprised of. The electric field **E** and magnetic field **H** inside the domain can be solved using Maxwell’s equation solvers such as the finite-difference-time-domain (FDTD) technique. The adjoint method computes the gradient  at all points within the design region.

The quantities involved in 3D simulations, such as the device permittivity  and FoM gradient , are often computed and stored as 3D matrices. Here, we find it convenient to flatten these into *N*-dimensional column vectors, where *N* is the total number of voxels in the device region. For the remainder of this derivation, all multidimensional quantities are considered *N*-dimensional vectors.

We define a metric to quantify how binary the device is. In this case, we use a simple absolute value function to define the binarization of a single pixel, whose mean across all pixels quantifies the binarization of the device. The absolute value function is scaled and shifted such that it evaluates to 1 at ϵ = ϵ_min_ and ϵ = ϵ_max_, and 0 at the permittivity midpoint, ϵ_mid_.
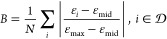
1
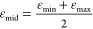
2

If we define , then the approximate change in binarization of the device given a permittivity step  is . This term can thus be constrained to predictably control the change in device binarization. We use an L1 norm to define the binarization here, since this is a simple function that provides equal weight to all permittivity values. The lack of differentiability at points where ϵ = ϵ_mid_ does not negatively affect the optimization as it might in other gradient-based techniques, and the derivative at ϵ_mid_ can simply be defined as the right- or left-hand derivative here. We comment on this further at the end of section 3 after exploring the proposed method’s behavior in an example.

Direct gradient ascent would shift the device permittivity in the direction of . However, the gradient may not point in a direction that increases the binarization of the device, so we reformulate the optimization to improve the FoM as much as possible while enforcing that the binarization increases by some amount β. This corresponds to solving the following constrained optimization problem, which we refer to as the suboptimization problem from now on since it is solved at every iteration of the main photonic optimization.
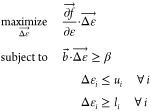
3

The vectors  and  control the maximum and minimum step sizes that the permittivity can undergo in a single iteration. They are also used to enforce the maximum and minimum permittivity constraints (i.e., ϵ_*i*_ ∈ [ϵ_min_, ϵ_max_] ∀*i*) since they can be set to 0 for a permittivity voxel at ϵ_min_ or ϵ_max_.

We convert eq 3 to a canonical form and set *c⃗* =  and *x⃗* = .
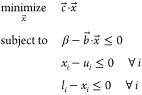
4

Since , there are 2*N* + 1 inequality constraints in eq 4 in addition to the *N* dimensions of the nonconstrained optimization problem. For large *N*, this problem is challenging to solve numerically even when using a technique such as simplex and interior-point methods that are intended for problems of this form. Considerable improvements in speed are obtained by reducing the multidimensional linear optimization to a single-dimensional nonlinear optimization, which can be done for this particular optimization regardless of the value of *N*. This is done by instead solving the Lagrange dual problem, which will have the same optimal point as eq 4 since the property of strong duality holds for linear optimizations.^17^ Further discussion of the computational complexities of various algorithms is available in the Supporting Information.

### 2.2 Solution

To obtain the dual problem, we start with the Lagrangian. This is defined as
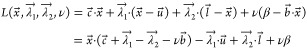
5

The dual function is defined as the infimum of the Lagrangian over :
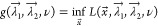
6

While the original optimization problem would be extremely difficult to solve computationally for large *N*, the solution to the dual function can be reduced down to a nonlinear optimization problem of only the scalar variable ν. This is derived in detail in the Supporting Information, with the following result:
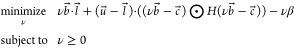
7

Here, ⊙ represents element-wise multiplication. The optimal solutions of the dual problem can be mapped back to original variable *x* using the Karush–Kuhn–Tucker (KKT) conditions, which are a set of conditions that hold under certain regularity conditions, of which linearity is sufficient. The instructions for this mapping are also given in the Supporting Information.

## 3 Results

This section provides example optimizations to illustrate the usage of the described technique and elaborate on several key points. The optimizations are performed on a 2D structure so that many optimizations can be run for analysis. We study device performance relative to a basic implementation of regular gradient ascent in which the permittivity is stepped in the exact direction of the gradient. We study this for three different cases of material-to-void refractive index contrast: a low index contrast of 1.5:1, a medium index contrast of 2.5:1, and a high index contrast of 3.0:1 (permittivity contrast 2.25:1, 6.25:1, and 9.0:1, respectively). Next, we pair this optimization technique with a level-set optimization that thresholds the continuous permittivity to a binary solution and reoptimizes the device with a level-set optimization to recover the lost performance from the threshold operation. Finally, we demonstrate how this technique can be used to control the overall ratio of material/void in the final device simply by shifting the binarization function in eq 1.

**Figure 1:**
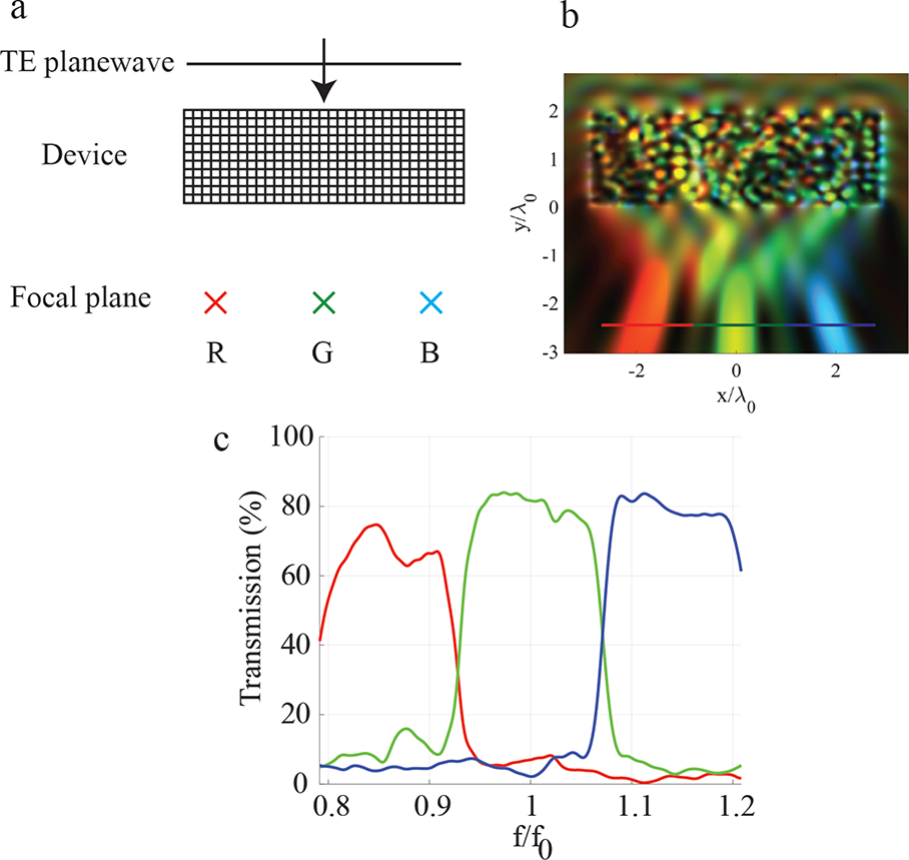
Example device. (a) A schematic of the device. The device is a spectral demultiplexer that focuses three equally spaced frequency bins to three distinct points marked in the focal plane. The example in this figure is a 2.5:1 refractive index contrast device (permittivity contrast 6.25:1). The input is a normally incident TE-polarized planewave. (b) The intensity |*E*|^2^ of each frequency is overlaid. Each frequency is drawn in its equivalent hue in the visible regime. (c) The power transmission of the output fields through the apertures. Each trace is drawn in the same color as the corresponding aperture drawn in (b).

The example optimization we use here is a spectral demultiplexer or “color splitter”.^4,9,18^Figure 1a illustrates the purpose of the device, which focuses three equally sized frequency bands of a normally incident TE plane-wave to three distinct points in the focal plane. The FoM function is defined as the intensity at the desired point in the focal plane, which allows us to use a simple dipole source for the adjoint source.^19^ When quantifying the performance of the device we use the power transmission through apertures centered at the dipole source. These apertures are drawn in Figure 1b as red, green, and blue horizontal lines in the focal plane. The gradients of every FoM (there is one FoM for each simulated frequency) are combined using a weighted average to obtain a single gradient vector, which is input as  in eq 3. This weighting procedure is described in detail in ref (18).

The device is optimized over a 42% fractional bandwidth, which is comparable to the fractional bandwidth of the visible spectrum. λ_0_ and *f*_0_ denote the center wavelength and frequency of the full bandwidth of the device. All FDTD simulations were conducted using Lumerical FDTD. In Figure 1b the intensity evaluated at the various frequencies are overlaid using their equivalent hue in the visible regime. The quantitative performance of a device (refractive index contrast 2.5:1) is shown in Figure 1c, which plots the power transmission into three monitors of size 1.56λ_0_, centered at the relevant focal points. Fabrication tolerance limits are not strictly enforced in this design, although the discretization of the geometry on a λ_0_/30 grid and the discretization of the FDTD simulation on a λ_0_/10*n*_max_ grid preclude arbitrarily small features in the design. After all optimizations, we simulated the final device on a finer grid to ensure that the FDTD results had properly converged. We noticed very little change to device behavior and efficiency after halving the simulation grid size in all directions. The results of this test is available in Figure S2.

### 3.1 Analysis of Hyperparameter β

In the optimization problem described by eq 3, the quantity β describes the amount that the device must binarize after stepping the permittivity. In our implementation we set this quantity to be a multiplicative factor (β_0_) of the max possible binarization (β_max_) during the current iteration. β_max_ is not constant during the optimization, since points on the device eventually reach a material boundary. The maximum and minimum allowable step size for each permittivity point in the device is contained in the vectors  and , respectively, which can be used to find β_max_. Thus, with the current implementation, the fixed optimization hyperparameter is β_0_ such that β = β_0_β_max_ at every iteration of the optimization.

In general, β_0_ controls how aggressively the device is binarized during the optimization. We are primarily interested in positive values of β_0_, since this is the case that forces the device to binarize by some positive amount each iteration. However, we will first make a note regarding the case of negative β values. If we were to compute a minimum change in binarization for a given iteration, denoted β_min_, which we could do using the same method that we use to compute β_max_ except taking the case that all voxels relax toward the center “gray” permittivity, then setting β ≤ β_min_ nullifies the binarization constraint. That is, the binarization constraint is satisfied no matter the permittivity step, so the solution will always be for each voxel to simply step in the direction of its own partial derivative with as large of a step as is allowed. In the case that β_min_ < β < 0, the permittivity steps are allowed to decrease the overall binarization to a limited extent.

We now look at the intended use case of β > 0 by studying the effects of the hyperparameter β_0_ from 0 to 1. We also show results for the case where β = 0, which represents the case where the binarization is not allowed to decrease, but also not forced to increase. This is a case that could be used to recover performance after a large transition in the device, which can occur for example in optimizations that employ sigmoidal filters that increase strength in discrete steps, without allowing the device to relax toward a gray solution.

**Figure 2:**
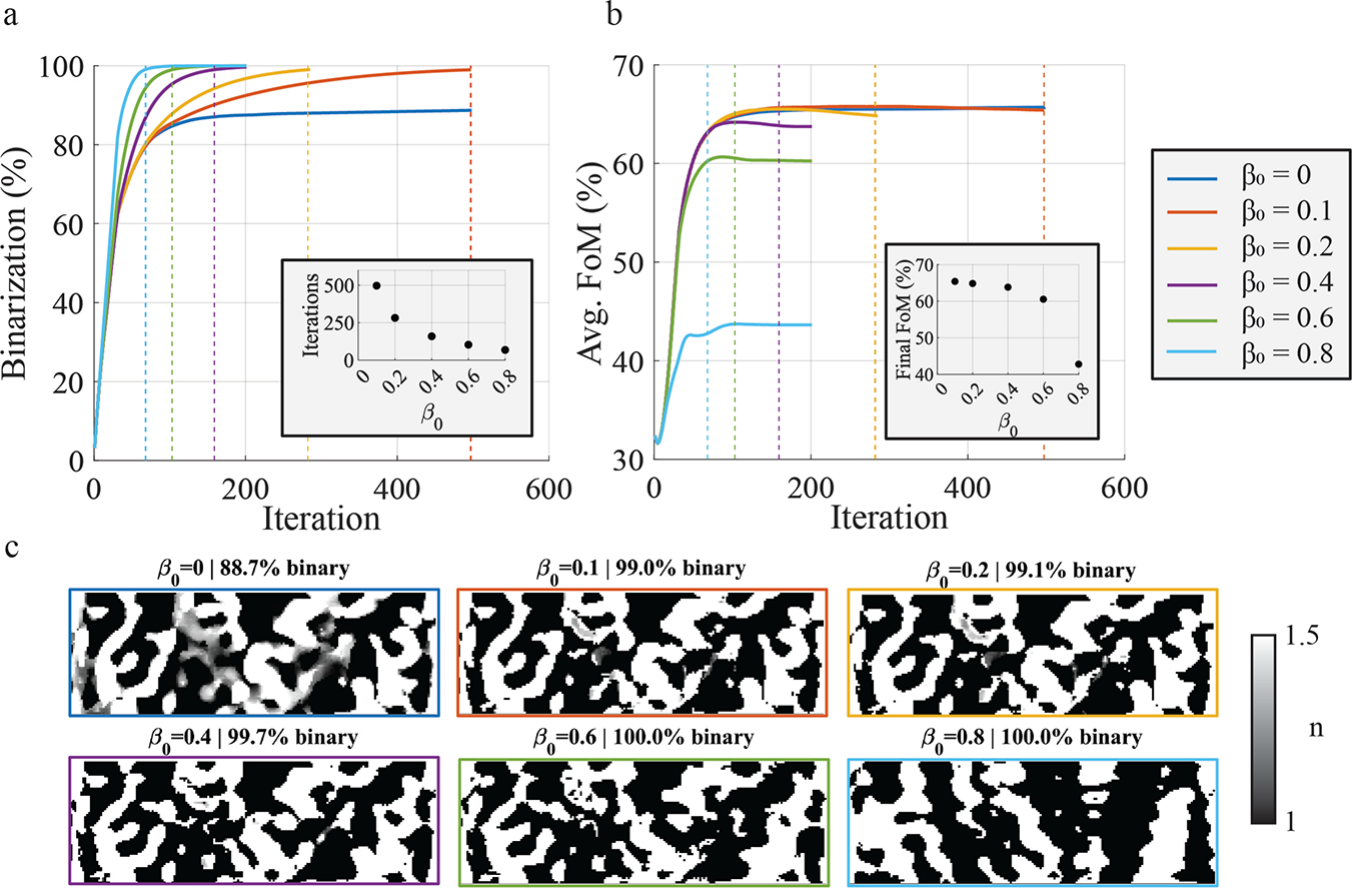
Analysis of the hyperparameter β_0_. (a) The evolution of device binarization for different values of β_0_. The dashed vertical lines represent when the optimization reached 99% binarization. The inset figure shows the number of iterations required to reach 99% binarization versus β_0_. (b) The evolution of the average FoM for different values of β_0_. The dashed vertical lines represent when the optimization reached 99% binarization. The inset figure shows the average FoM for each optimization at the point where they reached 99% binarization. (c) The index distribution of the final devices. The displayed index distribution corresponds to a device that was either optimized for 500 iterations or that reached 99% binarization, whichever came later. The exception is the β_0_ = 0, which is not guaranteed to converge to a binary solution and reached only 88.7% binarization after nearly 500 iterations.

For this example we study the optimization of a 1.5:1 refractive index contrast (permittivity 2.25:1) spectral demultiplexer and use a max permittivity step size of ±0.02 each iteration. This quantity was found by running a few iterations to roughly compute the largest step size that does not cause instability in the optimization. After choosing this step size, all optimizations were run only once to obtain the results shown. Optimizations were performed until the device was 99% binary or for 500 iterations (whichever came first) using the algorithm summarized in Algorithm 1. Figure 2a shows the evolution of the binarization during the optimizations, and confirmed the expected result that larger β_0_ values binarize the device faster. The number of iterations required to reach 99% binarization is shown in the subset of Figure 2a. The case of β = 0 was also shown here, but since β_0_ is not strictly positive, the device is not guaranteed to converge to a binary solution. After nearly 500 iterations, the binarization in the β_0_ = 0 case had stagnated at 88.7%.

**Figure:**
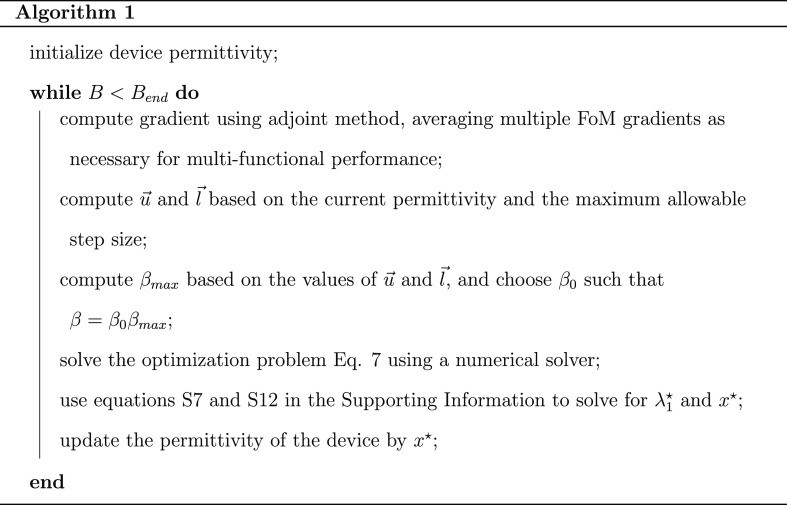


Larger values of β_0_ tend to converge to lower performing solutions, which can be seen from the traces in Figure 2b. This suggests a trade-off between optimization time (by way of the aggressiveness of the binarization constraint) and the performance of the converged device, although there is always the theoretical possibility of a more aggressive optimization converging to a better local solution by chance. The final performance of each device, defined here as the performance of the device when it first passes 99% binarization, is shown as a function of β_0_ in the subset of Figure 2b. The index distribution of each device at its final iteration is shown in Figure 2c. In this case, the final device performance monotonically decreases with β_0_. In particular, a sharp decrease in performance was observed when moving from β_0_ = 0.6 to β_0_ = 0.8, suggesting that the β_0_ = 0.8 excessively prioritizes binarizing the device over improving performance. We did not study the case of β_0_ = 1 because this does not represent a useful case. In this case, the first iteration of the optimization has the freedom to step in the direction of the gradient, and all subsequent iterations are forced to step every voxel toward its nearest boundary. Thus, it is equivalent to stepping the permittivity once in the direction of the gradient then thresholding the device, which in general will not yield useful devices.

### 3.2 Incorporating Projection Filters and Level-Set Finalization

A common technique in inverse-design involves optimizing for an auxiliary variable  whose relationship to the permittivity is , where *S* is a projection filter or compound function of projection filters.^20^ A common filter used to encourage binary designs is the sigmoid function, shown in eq 8.
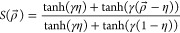
8

Projection filters can be included in the optimization technique presented in this work by recasting eq 3 in terms of  by way of the chain rule. Denoting  as the derivative of *S* with respect to , the new optimization problem is eq 9.
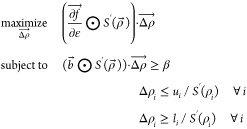
9We compare our method, which we refer to here as modified gradient ascent, with and without a sigmoid projection filter for different refractive index contrasts. The contrasts studied here are 1.5:1, 2.5:1, and 3.0:1 (in terms of permittivity ϵ: 2.25:1, 6.25:1, and 9.0:1). We use β_0_ = 0.2 and a maximum and minimum permittivity step size of ±0.02 for both cases. In the sigmoid case, the strength of the sigmoid (γ) begins at 0.1 and is multiplied by a factor of 2 at various points during the optimization. The length of each epoch is 30, 50, and 100 iterations for the 1.5:1, 2.5:1, and 3.0:1 index contrast examples, respectively. The sigmoid threshold η in eq 8 is 0.5 for all cases.

We also include a basic implementation of gradient ascent for comparison, which simply steps the device permittivity in the exact direction of the electromagnetic FoM gradient (i.e., a steepest ascent optimization). The permittivity update is computed with , where α controls the specific step size. To ensure both stability and acceptable convergence, we dynamically alter α such that the voxel with the largest absolute value of gradient is stepped by a permittivity of ±0.1. Like the previous examples, this quantity was found by running a few iterations to roughly compute the largest step size that does not cause instability in the optimization. The step size for this procedure is a factor of 5 larger than the step size used in the modified gradient ascent method. The need for a smaller step size in the modified method is likely due to all device voxels being perturbed by the same amount at every iteration, whereas for the direct gradient ascent method only the voxel with the largest gradient is perturbed by the largest possible step size.

**Figure 3:**
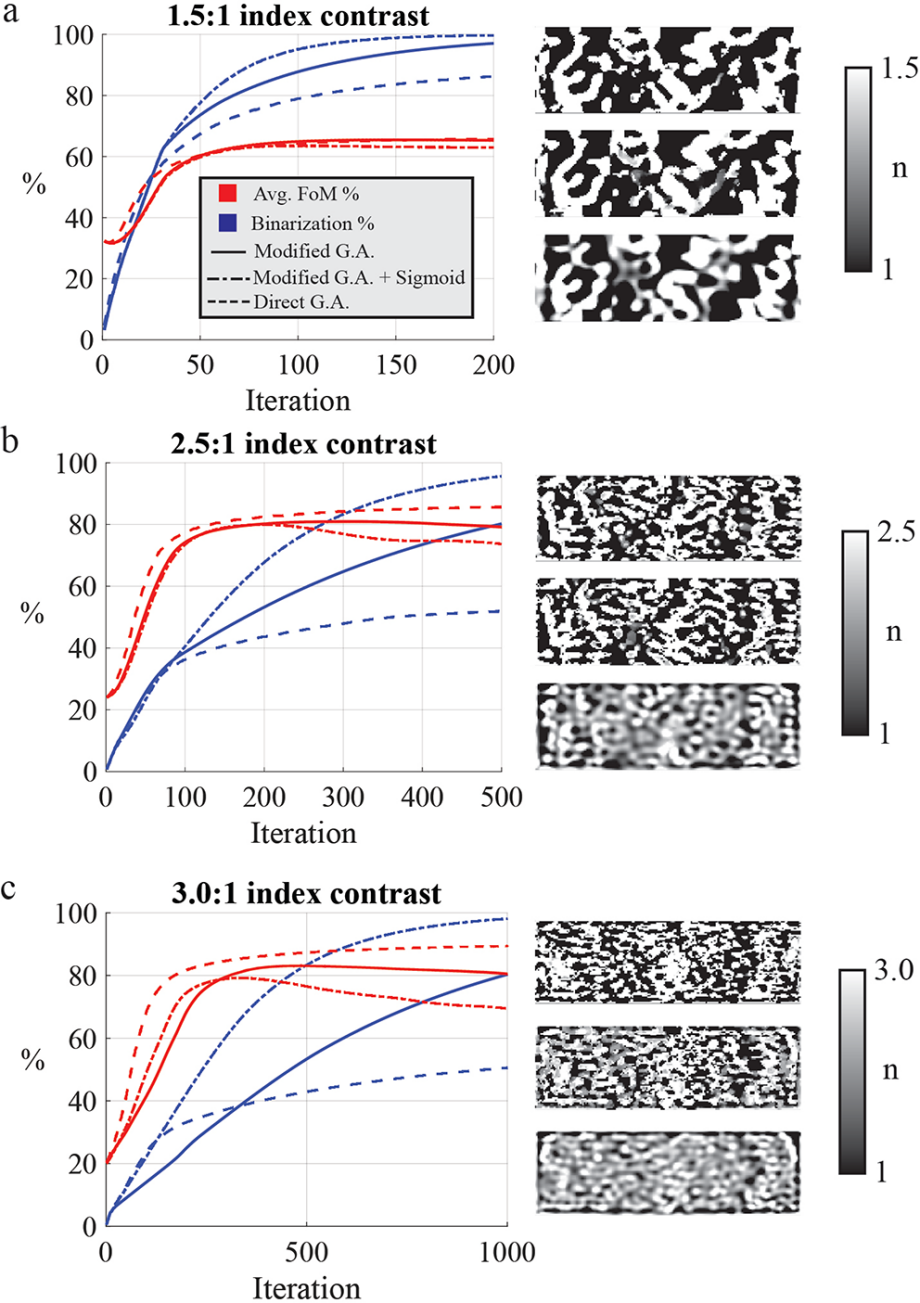
FoM and binarization convergence curves for different index contrasts. The FoM (averaged across all functionalities) and binarization are drawn as red and blue traces, respectively. The modified gradient ascent (G.A.), modified G.A. with a sigmoid projection filter, and direct G.A. methods are drawn as solid, dot-dashed, and dashed lines, respectively. The final index profile of each device is shown as a greyscale colormap. In each subset, the top device is optimized with the modified G.A. algorithm with a sigmoid filter, the middle is optimized with the modified G.A. without a sigmoid filter, and the bottom is optimized with the direct G.A. procedure. The index contrasts are (a) 1.5:1, (b) 2.5:1, and (c) 3.0:1.

The results for the different refractive index contrasts are shown in Figure 3. The plotted average FoM in Figure 3a–c is the transmission through the desired aperture averaged over all frequencies. In the low contrast case (1.5:1) in Figure 3a, the optimizations perform equally well in terms of FoM. In the medium contrast case (2.5:1) in Figure 3b, the direct gradient ascent optimization converges to a final FoM value of 84% while the binarization stagnates at 54%. This implies that optimization has found a local maximum of the FoM where the device is not binary. This type of solution is forbidden in the modified gradient ascent approach. In fact, the FoM reaches a maximum near iteration 200 in the sigmoid case and 300 in the nonsigmoid, but continues to search for a solution that is binary in order to satisfy the binarization constraint. It is necessary that the maximum FoM of a binarized device is less-than-or-equal to the maximum FoM of a greyscale device, since the former is a subset of the latter. Thus, when using this method a decreasing FoM at some point in the optimization is often expected, and is conceptually similar to discretely increasing a binary regularization parameter (e.g., sigmoid strength) to force a solution to be binary. However, in the latter case, knowledge of the gradient is not used as information while increasing the binarization.

The difference between the direct and modified gradient methods becomes yet more apparent in the highest index contrast case (3.0:1) in Figure 3c. In this case, the direct gradient ascent optimization converges to a 48% binary device after 1000 iterations, while the modified gradient ascent optimization reaches 98% by that point when employing a sigmoid filter, and 80% without a sigmoid filter. In all cases, including a sigmoid projection filter improves the convergence speed of the modified gradient ascent method.

While the modified gradient ascent approach will eventually converge to a 100% binary device simply because any other solution is forbidden, waiting for a binary solution is not strictly required. Instead, the optimization can be concluded before the device is 100% binary, thresholded to the nearest material boundary, and then used as an initial seed to a level-set optimization. Level-set optimizations tend to be dependent on initial seed,^11^ and we study the dependence of the performance of a level-set optimization with respect to the binarization percentage of an initial seed obtained with the modified gradient ascent approach. For this study, we use an implementation of level-set optimization based on signed distance functions.^21^ This implementation does not incorporate topological derivatives to facilitate the nucleation of holes or bridging of gaps, which can increase the robustness of level-set implementations.

**Figure 4:**
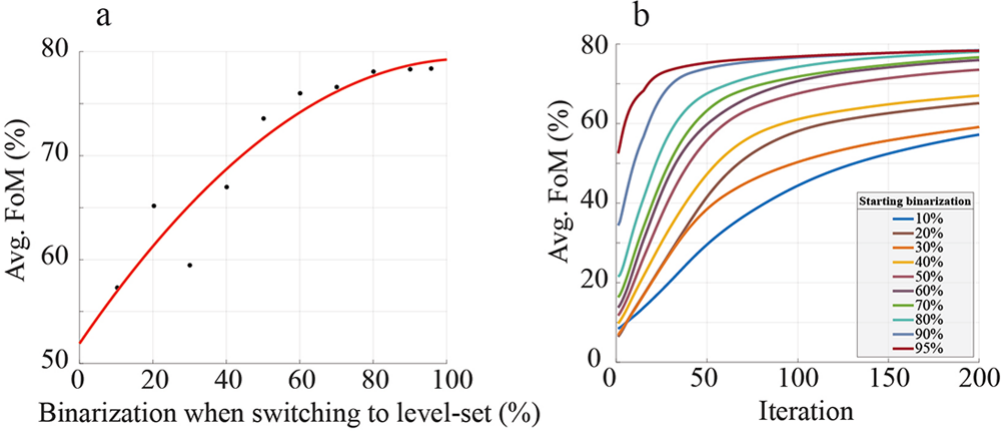
Combining the continuous density-based optimization with a level-set optimization. (a) The level-set optimization uses an initial seed given by the results of the continuous density-based optimization at different starting binarization values. The average FoM after 200 iterations of level-set optimization is plotted as a function of this starting binarization. The red curve is a quadratic fit. (b) The convergence curves of each level-set optimization.

Figure 4a shows the converged average FoM of a 2.5:1 medium index contrast device as a function of the binarization percentage of the initial seed and Figure 4b shows the convergence curves for the individual level-set optimizations. The results suggest that the level-set optimization does indeed depend on initial seed, and that in general the more binarized starting seeds yield a better level-set optimized device. Beyond 40% binarization, the performance monotonically increased with starting binarization, however the diminishing gains suggest that terminating the density-based optimization around 80% will yield nearly optimal performance while minimizing the time spent in the density-based phase of the optimization.

### 3.3 Controlling the Overall Material/Void Fraction

In topology optimization, the need occasionally arises to control the overall material/void fraction,^22^ and the exact method for achieving this depends on the optimization algorithm. For the method described in this paper, the definition of the binarization function can be shifted with respect to permittivity in order to converge to devices with different overall material/void ratios. Equation 1 uses the midpoint of the two material permittivities as the center of the absolute value function. We can introduce a shift term, δϵ:
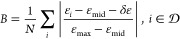
10

Intuitively, the binarization constraint in eq 3 can be thought of as defining whether a permittivity point should increase or decrease in order to binarize the device more, depending on its current value of permittivity. In eq 3 (with no shift about the midpoint), points that are above the midpoint are encouraged to increase further since this is the direction of binarization, and similarly points below the midpoint are encouraged to decrease further. Shifting the cusp away from the midpoint affects how the permittivity is encouraged to become more binary. In general, shifting the cusp of *B* to the left (positive δϵ) yields a device with more material and vice versa. This can be a useful feature for ensuring mechanical robustness, reducing the amount of a lossy material or as a tunable hyperparameter for finding different local optima.

**Figure 5:**
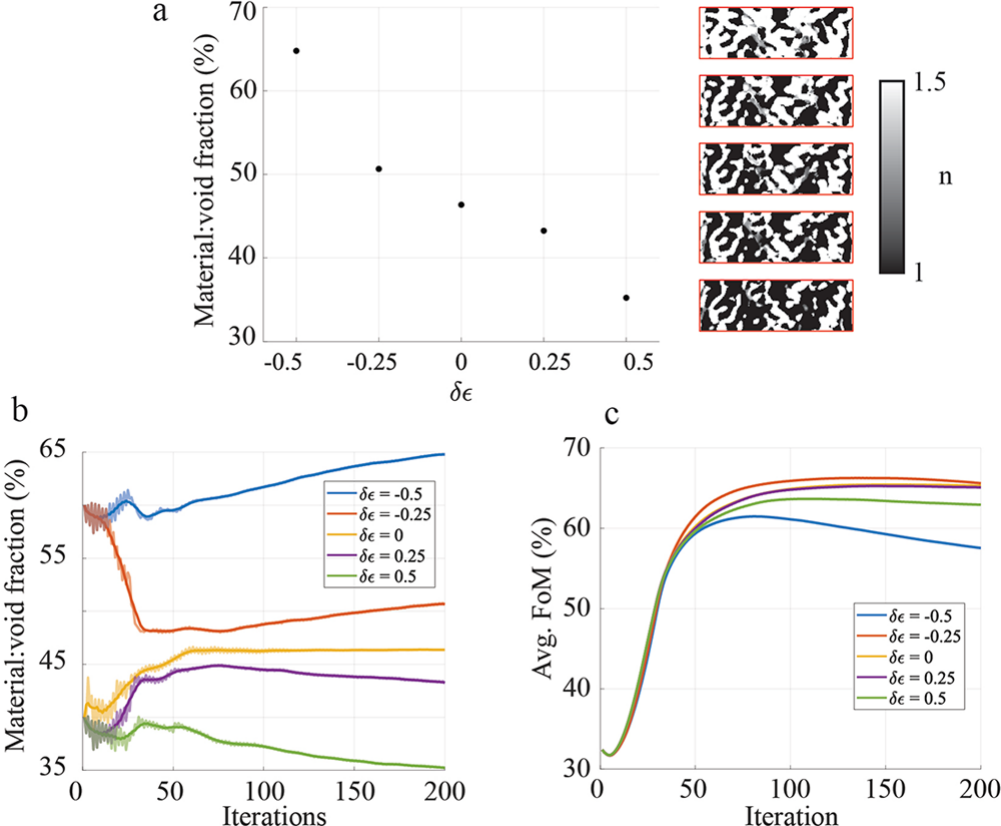
Controlling the overall fraction of material. (a) The material-to-void ratio for different shifts in the definition of binarization. To the right of the plots is a picture of the resulting optimized device, where white is material and black is void. From top to bottom the devices correspond to δϵ = −0.5, −0.25, 0, 0.25, and 0.5. (b) The evolution of the material-to-void ratio during the optimization. Each traces shows the raw data and a five-iteration rolling average. (c) The evolution of the FoM during the optimization.

This technique is demonstrated in a low index contrast (1.5:1) device. Figure 5a shows the final material/void fraction as a function of δϵ, which is varied from −0.5 to +0.5 about the midpoint permittivity of 1.625. The resulting material distribution is also shown for each optimized device. Figure 5b shows the evolution of the material/void fraction over the course of the optimization for the different δϵ values. The largest change in material:void fraction tends to occur in the early stages of optimization in this example. Figure 5c shows the evolution of the average FoM for different values of δϵ. The devices with the worst final performance are the ones with the most extreme δϵ, with the device consisting of primarily material being substantially worse than the rest. The best performing device was optimized with δϵ = −0.25. Although not demonstrated here, it is also possible that δϵ can be actively tuned during the optimization to approach a desired material/void ratio set point.

It was previously mentioned that the binarization definition lacks differentiability at ϵ = ϵ_mid_, and we claimed that this does not negatively affect the optimization. To elaborate, this will only affect voxels whose permittivity is within one step size of the ϵ_mid_. Of these voxels, the ones chosen to move toward ϵ_mid_ will do so to increase device performance, and the effect of crossing over the cusp of at ϵ_mid_ will only cause the binarization to increase more than expected, and thus the optimization constraints remain satisfied. It is worth noting that the effect of choosing a left- or right-hand derivative at this point will have an effect on the bias toward one material or the other, particularly if the device optimization begins with all voxels at ϵ_mid_ with a high value of β_0_. To avoid this case, β_0_ can be set to 0 for the first several iterations to remove any initial bias toward a particular material boundary.

## 4 Conclusion

We have presented a suboptimization technique that is constrained to converge to fully binary solutions. The technique involves solving a suboptimization at each iteration that maximizes the change in device performance while forcing the step in dielectric permittivity to increase the overall binarization of the device by a certain amount. This technique is computationally infeasible to solve directly with a numerical solver, but the optimization problem is reduced to a one-dimensional nonlinear optimization problem by instead solving the Lagrange dual problem.

The usefulness of the technique becomes apparent when optimizing devices with a large refractive index contrast between the two material boundaries, where traditional gradient-based techniques tend to stagnate at nonbinary solutions. It is shown that our level-set optimization depends on the initial seed, as has been discussed in literature over the years, and that beginning the level-set optimization with more binary seeds leads to better final device performance. Lastly, we showed an example where the material-to-void fraction of the optimized device can be controlled by simply shifting the definition of binarization.

The specific suboptimization presented here was intended to constrain the device only to binary solutions. However, there were no assumptions made on the binarization function *B* other than the existence of a derivative with respect to ϵ. Thus, this function can describe other properties of the device and the optimization procedure can be used to constrain other device properties in future works. A potentially promising function to constrain with this method would be a net transmission FoM, effectively forcing the optimization to converge only to highly transmissive solutions. This would be particularly useful if the same device can be cascaded to improve performance, such as for a bandpass filter in which case a highly transmissive passband is extremely important.

## Supporting Information Available

Details regarding the derivation of eq 7, the computational complexity of the algorithm, a MATLAB example for computing this computational complexity, and convergence plots for the optimizations shown in Figure 3 (PDF)
