# Supplementary material for: Constraining Continuous Topology Optimizations to Discrete Solutions for Photonic Applications
Source: ACS Photonics. 2023 Jan 9;10(4):836–44. doi: 10.1021/acsphotonics.2c00862 (PMC10119985; doi:10.1021/acsphotonics.2c00862)
Supplement: Supplementary file 1 — ph2c00862_si_001.pdf [file ph2c00862_si_001.pdf]

Constraining continuous topology optimizations to discrete solutions for photonic applications: supplemental document

Conner Ballew, Gregory Roberts, Tianzhe Zheng, and Andrei Faraon

1 Derived solution to Lagrange dual problem from main text

To obtain the dual problem, we start with the *Lagrangian*. This is defined as:

$$\begin{aligned} L(\vec{x}, \vec{\lambda_1}, \vec{\lambda_2}, \nu) &= \vec{c} \cdot \vec{x} + \vec{\lambda_1} \cdot (\vec{x} - \vec{u}) + \vec{\lambda_2} \cdot (\vec{l} - \vec{x}) + \nu(\beta - \vec{b} \cdot \vec{x}) \\ &= \vec{x} \cdot (\vec{c} + \vec{\lambda_1} - \vec{\lambda_2} - \nu\vec{b}) - \vec{\lambda_1} \cdot \vec{u} + \vec{\lambda_2} \cdot \vec{l} + \nu\beta \end{aligned} \tag{S1}$$

The *dual function* is defined as the infimum of the Lagrangian over $\vec{x}$:

$$g(\vec{\lambda_1}, \vec{\lambda_2}, \nu) = \inf_{\vec{x}} L(\vec{x}, \vec{\lambda_1}, \vec{\lambda_2}, \nu) \tag{S2}$$

Since (S1) is linear, the infimum is $-\infty$ if any slope is non-zero.

$$g(\vec{\lambda_1}, \vec{\lambda_2}, \nu) = \begin{cases} -\infty & \vec{c} + \vec{\lambda_1} - \vec{\lambda_2} - \nu\vec{b} \neq \vec{0} \\ -\vec{\lambda_1} \cdot \vec{u} + \vec{\lambda_2} \cdot \vec{l} + \nu\beta & \vec{c} + \vec{\lambda_1} - \vec{\lambda_2} - \nu\vec{b} = \vec{0} \end{cases} \tag{S3}$$

The optimization we wish to solve is called the *Lagrange dual problem*:

$$\begin{aligned} \underset{\vec{\lambda_1}, \vec{\lambda_2}, \nu}{\text{maximize}} \quad & g(\vec{\lambda_1}, \vec{\lambda_2}, \nu) \\ \text{subject to} \quad & \vec{\lambda_1} \geq 0, \\ & \vec{\lambda_2} \geq 0, \\ & \nu \geq 0 \end{aligned} \tag{S4}$$

Incorporating (S3) into Eq. S4, we get:

$$\begin{aligned} \underset{\vec{\lambda_1}, \vec{\lambda_2}, \nu}{\text{maximize}} \quad & -\vec{\lambda_1} \cdot \vec{u} + \vec{\lambda_2} \cdot \vec{l} + \nu\beta \\ \text{subject to} \quad & \vec{\lambda_1} \geq 0, \\ & \vec{\lambda_2} \geq 0, \\ & \nu \geq 0, \\ & \vec{c} + \vec{\lambda_1} - \vec{\lambda_2} - \nu\vec{b} = 0 \end{aligned} \tag{S5}$$

The fourth constraint of (S5) can be used to write the FoM and the second constraint in terms of only ν and λ_1.

$$\begin{aligned} \underset{\vec{\lambda_1}, \nu}{\text{maximize}} \quad & -\vec{\lambda_1} \cdot \vec{u} + (\vec{c} + \vec{\lambda_1} - \nu\vec{b}) \cdot \vec{l} + \nu\beta \\ \text{subject to} \quad & \vec{\lambda_1} \geq 0, \\ & \vec{\lambda_1} \geq \nu\vec{b} - \vec{c}, \\ & \nu \geq 0 \end{aligned} \tag{S6}$$

For any ν, the optimal elements of $\vec{\lambda_1}$ are as small as possible since $\vec{u} \geq 0$ and $\vec{l} \leq 0$. The solution to $\vec{\lambda_1}$ is obtained by satisfying the constraints while minimizing $\vec{\lambda_1}$. The solution for the i-th element of $\vec{\lambda_1}$ is thus:

$$\lambda_{1,i} = \begin{cases} 0 & \nu b_i - c_i \leq 0 \\ \nu b_i - c_i & \nu b_i - c_i > 0 \end{cases} = (\nu b_i - c_i) H(\nu b_i - c_i) \tag{S7}$$

where $H(x)$ is the Heaviside step function. $\vec{\lambda_2}$ is solved using the fourth constraint of (S5).

$$\vec{\lambda_2} = \vec{c} + \vec{\lambda_1} - \nu\vec{b} = \vec{c} + (\nu\vec{b} - \vec{c}) \odot H(\nu\vec{b} - \vec{c}) - \nu\vec{b} \tag{S8}$$

Here, $H(\vec{x})$ represents the Heaviside function applied element-wise to $\vec{x}$ and $\odot$ represents element-wise multiplication. Since the optimal $\vec{\lambda_1}$ and $\vec{\lambda_2}$ are dependent on ν, the dual problem is a function of only $\nu \in \mathbb{R}$. The optimization can be reduced to a single dimension as follows.

$$\begin{aligned} \underset{\nu}{\text{minimize}} \quad & \nu\vec{b} \cdot \vec{l} + (\vec{u} - \vec{l}) \cdot ((\nu\vec{b} - \vec{c}) \odot H(\nu\vec{b} - \vec{c})) - \nu\beta \\ \text{subject to} \quad & \nu \geq 0 \end{aligned} \tag{S9}$$

Note that we have converted this from a maximization problem to a minimization problem, and omitted a $-c \cdot l$ term since it is constant. This problem can be solved numerically using open-source functions. We denote the optimal dual variables as $(\nu^\star, \vec{\lambda_1^\star}, \vec{\lambda_2^\star})$. The optimal solutions of the dual problem can be mapped back to original variable x using the *Karush–Kuhn–Tucker (KKT) conditions*, which are a set of conditions that hold under certain regularity conditions. Fortunately, linearity of the constraints is a sufficient condition for the KKT conditions to hold. We use the KKT condition of *complementary slackness* to translate $(\nu^\star, \vec{\lambda_1^\star}, \vec{\lambda_2^\star})$ to the optimal $\vec{x}$ that we call $\vec{x^\star}$.

$$\vec{\lambda_1^\star} \odot (\vec{x^\star} - \vec{u}) = \vec{0} \tag{S10}$$

$$\vec{\lambda_2^\star} \odot (\vec{l} - \vec{x^\star}) = \vec{0} \tag{S11}$$

The solution is

$$x_i^\star = \begin{cases} u_i & \lambda_{1,i} \neq 0 \\ l_i & \lambda_{1,i} = 0 \end{cases} \tag{S12}$$

2 Computational complexity

The Matlab code in Section 4 can be used to evaluate the computational complexity of dual-simplex, interior-point, and the method used in this manuscript. The analysis was done on a computer with the following specifications:

- PC: AMD Ryzen Threadripper 3990X 64-Core Processor 2.90 GHz
- RAM: 128 GB (4x32 GB, 3200 MHz DDR4)
- GPU: GeForce RTX 3070
- OS: Windows 10 (21H2)
- MATLAB version: R2020B Update 7

Figure S1 shows the computation time for each algorithm as a function of the input dimension size. Similar plots were obtained with Python's SciPy library, and code for this is available on request.

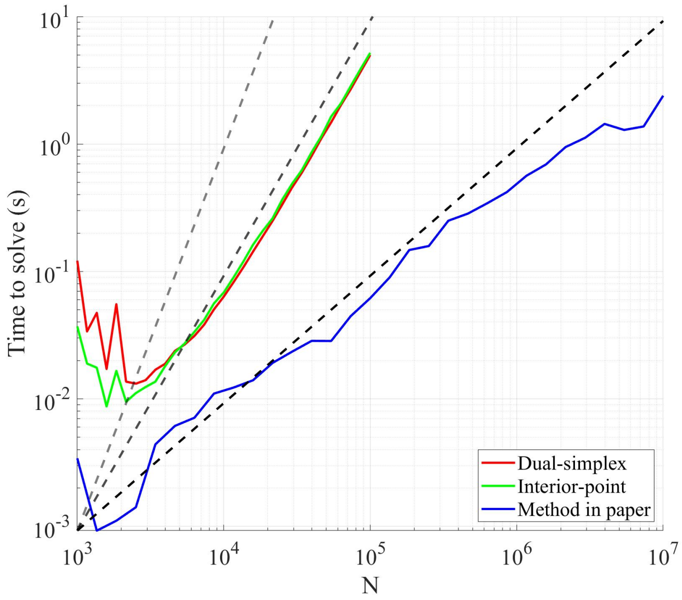

Figure S1: **Computational complexity of various algorithms that can be used to solve Eq. 3 in the main manuscript. The red, green, and blue lines plot the computation time with respect to the number of dimensions when using dual-simplex, interior-point, and the method described in Section 1. The grey dashed lines are representative of N, N^2, and N^3 scaling.**

3 Convergence tests

Optimizations performed in the main manuscript used a $\lambda_0/(10 \times n_{max})$ FDTD grid resolution, where λ_0 is the center wavelength of the optimized band and n_{max} is the maximum refractive index that the device voxels can assume (which varies from 1.0 to 3.0 in the main manuscript). To confirm that the final results are realistic, we simulate the final structure on a finer grid of $\lambda_0/(20 \times n_{max})$. The transmissions through the three different apertures of the focal plane are drawn in Fig. S2, similar to how the transmissions were plotted in Fig. 1 of the main manuscript.

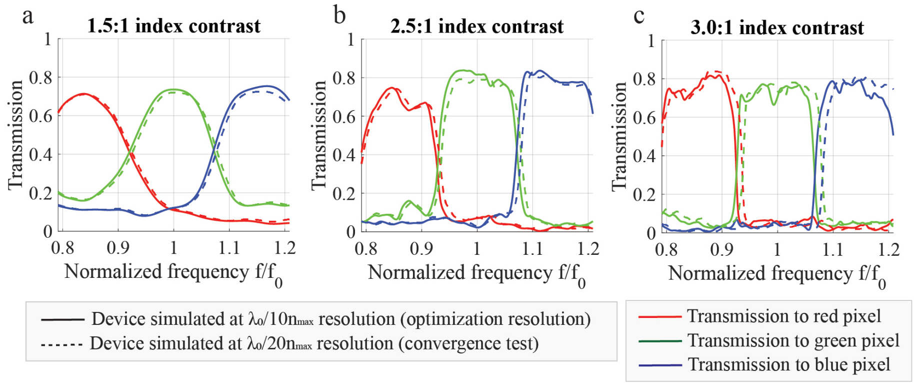

Figure S2: **Convergence plots for the final devices of Fig. 3 in the main manuscript.**

4 MATLAB computational complexity code

```
% Set a random seed to obtain same plots as shown
rng(42);

% Number of dimensions to simulate
N_regular_methods = round(logspace(3,5,31)); % N=6 takes a couple hundred seconds
N_paper_method = round(logspace(3,7,31));

% Paper method's hyperparameter. First constraint of Eq. 3.
beta_0 = 0.2;
max_density_step = 0.03;

% 'Binarization' function. Eq. 1 in main manuscript.
B = @(vals, N) (1/N) * sum(abs( (vals - 0.5) ./ 0.5 ));

% Optimality and constrain tolerances of dual-simplex and interior-point
% methods.
eps = 1E-6;
eps_constraint = 1E-6;

% Simulates dual-simplex and interior point methods with dimensions in
% N_regular_methods. The paper's method is also simulated, and the
% resulting solution is directly compared with the regular methods to show
% the solutions are nearly the same.
for i=1:length(N_regular_methods)
    N_curr = N_regular_methods(i);

    % Generate random density and gradient values
    values = rand([N_curr,1]);
    rand_gradient = rand([N_curr,1]) - 0.2;

    % Compute b, the derivative of B.
    b = 2*sign(values - 0.5) / N_curr;

    % Compute the largest possible step up for all values
    up_step = values + max_density_step;
    up_step(up_step > 1) = 1;
    u = up_step - values;

    % Compute the largest possible step down for all values
    down_step = values - max_density_step;
    down_step(down_step < 0) = 0;
    l = down_step - values;

    % Find max possible change in binarization
    values_test = values;
    values_test(values_test < 0.5) = values_test(values_test ≤ 0.5) + l(values_test ≤ 0.5);
    values_test(values_test > 0.5) = values_test(values_test > 0.5) + u(values_test > 0.5);
    B_max_change = B(values_test, N_curr) - B(values, N_curr);
    beta = beta_0*B_max_change;

    % Dual simplex
    options = optimoptions('linprog','Algorithm','dual-simplex', 'OptimalityTolerance', eps, ...
        'ConstraintTolerance', eps_constraint);
    tic;
    x_dual = linprog(-rand_gradient,-b',-beta,[],[],l,u, options);
    t1(i) = toc;

    % Interior point
    options = optimoptions('linprog','Algorithm','interior-point', 'OptimalityTolerance', ...
        eps, 'ConstraintTolerance', eps_constraint);
    tic;
    x_int_pt = linprog(-rand_gradient,-b',-beta,[],[],l,u, options);
    t2(i) = toc;
```

```
    % Paper method
    options = optimset('TolX',1E-4,'TolFun',1E-4);
    c = -rand_gradient;
    f = @(nu) (nu*dot(b,l) + dot((u-l), (nu.*b-c).*heaviside(nu.*b-c)) - nu*beta);
    nu_opt = fminbnd(f,0,1E6,options);

    lamb1 = (nu_opt.*b - c).*heaviside(nu_opt.*b - c);
    x = u.*ones(size(values));
    x(lamb1 == 0) = l(lamb1 == 0);

    % Compare accuracy of approaches relative to one another
    agreement_int_pt(i) = sum((x>0) == (x_int_pt > 0)) / N_curr;
    agreement_dual_simplex(i) = sum((x>0) == (x_dual > 0)) / N_curr;
end

% Simulates just the paper method. This loop can go to much higher
% dimension counts without crashing PC.
for i=1:length(N_paper_method)
    N_curr = N_paper_method(i);

    % Generate random density and gradient values
    values = rand([N_curr,1]);
    rand_gradient = rand([N_curr,1]) - 0.2;

    % Compute b, the derivative of B.
    b = 2*sign(values - 0.5) / N_curr;

    % Compute the largest possible step up for all values
    up_step = values + max_density_step;
    up_step(up_step > 1) = 1;
    u = up_step - values;

    % Compute the largest possible step down for all values
    down_step = values - max_density_step;
    down_step(down_step < 0) = 0;
    l = down_step - values;

    % Find max possible change in binarization
    values_test = values;
    values_test(values_test < 0.5) = values_test(values_test ≤ 0.5) + l(values_test ≤ 0.5);
    values_test(values_test > 0.5) = values_test(values_test > 0.5) + u(values_test > 0.5);
    B_max_change = B(values_test, N_curr) - B(values, N_curr);
    beta = beta_0*B_max_change;

    % Paper method
    options = optimset('TolX',1E-4,'TolFun',1E-4);

    tic;
    c = -rand_gradient;
    f = @(nu) (nu*dot(b,l) + dot((u-l), (nu.*b-c).*heaviside(nu.*b-c)) - nu*beta);
    nu_opt = fminbnd(f,0,1E6,options);

    lamb1 = (nu_opt.*b - c).*heaviside(nu_opt.*b - c);
    x = u.*ones(size(values));
    x(lamb1 == 0) = l(lamb1 == 0);
    t3(i) = toc;
end

% Results
disp("Agreement with dual-simplex method: " + ...
    num2str(100*agreement_int_pt(length(agreement_int_pt))) + "%");
disp("Agreement with interior point method: " + ...
    num2str(100*agreement_int_pt(length(agreement_int_pt))) + "%");

%%
figure;
hold on;
```

```
% Draw the computation time dats
h1=loglog(N_regular_methods,t1, 'Color', 'r', 'LineStyle', '-', 'DisplayName', 'Dual-simplex');
h2=loglog(N_regular_methods,t2, 'Color', 'g', 'LineStyle', '-', 'DisplayName', 'Interior-point');
h3=loglog(N_paper_method,t3, 'Color', 'b', 'LineStyle', '-', 'DisplayName', 'Method in paper');
set(gca,'YScale','log','XScale','log')
yl = ylim ;
xl = xlim;

% Draw representative lines of N, N^2, and N^3 scaling.
p=loglog(N_paper_method, yl(1).*(N_paper_method./xl(1)), 'Color', 'k', 'LineStyle', '--');
p.Color(4) = 1;
p=loglog(N_paper_method, yl(1).*(N_paper_method./xl(1)).^2, 'Color', 'k', 'LineStyle', '--');
p.Color(4) = 0.7;
p=loglog(N_paper_method, yl(1).*(N_paper_method./xl(1)).^3, 'Color',  'k', 'LineStyle', '--');
p.Color(4) = 0.5;

xlim(xl);
ylim(yl);
grid on;

legend([h1,h2,h3], 'Location', 'SouthEast');

xlabel('N');
ylabel('Time to solve (s)');
```
